# Radical radiotherapy for cervical cancer and meningioma with a history of Guillain-Barré syndrome: A case report

**DOI:** 10.1097/MD.0000000000032124

**Published:** 2022-12-02

**Authors:** Kangli Deng, Yanxin Yu, Liben Ge, Kangyan Deng, Mo Zhang

**Affiliations:** a Department of Health Management & Institute of Health Management, Sichuan Provincial People’s Hospital, University of Electronic Science and Technology of China, Chengdu, China; b Department of Oncology, Chengdu 363 Hospital Affiliated to Southwest Medical University, Chengdu, China; c Department of radiotherapy, Jilin Provincial People’s Hospital, Changchun, China; d Department of Gynecology, Langzhong People’s Hospital, Langzhong, China.

**Keywords:** cervical cancer, GBS, meningioma, radical radiotherapy, SRS

## Abstract

Guillain-Barré syndrome (GBS) is a rare autoimmune disease. Patients with cervical malignancies and intracranial meningiomas after the course of GBS are even rarer. There are no relevant reports at present.

**Patient concerns:**

We report a patient who developed cervical cancer (CC) and intracranial meningioma simultaneously after the course of GBS.

**Diagnoses:**

The history, pelvic enhanced magnetic resonance imaging (MRI) and pathology confirmed cervical squamous cell carcinoma, and the head enhanced MRI confirmed meningioma.

**Intervention:**

After multi-disciplinary team, the patient received head stereotactic radiosurgery for meningioma and radical radiotherapy for CC.

**Outcomes:**

The follow up for 1 year after treatment revealed a complete remission of the cervical tumor, stable disease of the meningioma, and no signs of GBS recurrence. Up to now, the general condition of the patient is acceptable and the curative effect is satisfactory.

**Lessons:**

This case report aims to improve the oncologists’ understanding of malignant tumors with rare autoimmune diseases and provide treatment reference for similar diseases in the future.

## 1. Introduction

Guillain-Barré syndrome (GBS), also known as acute infectious polyneuritis, is a rare but fatal autoimmune disease caused by an autoimmune dysfunction. It is characterized by the demyelination of the peripheral nerves and nerve roots and by inflammatory response of lymphocytes and macrophages around the small vessels. Brain nerves and respiratory muscles are often involved, and secondary axonal degeneration can occur in severe cases.^[1]^ The clinical features are symmetrical flaccid paralysis of the limbs, cranial nerve damage and protein separation in the cerebrospinal fluid. The treatment of this disease includes symptomatic support therapy, immunoglobulin therapy, plasma exchange therapy, and hormone therapy. The recurrence rate of GBS can be as high as 6%, and most studies showed a rate between 2% and 6.8%,^[2,3]^and this is called recurrent GBS. Recurrent patients have rapid symptom progression^[4]^ and long onset time, they are difficult to be cured and show a slow recovery. This disease is usually triggered by Campylobacter jejuni, viral infection (such as cytomegalovirus and EB virus), surgery or trauma.^[5]^

Cervical cancer is one of the most common malignant tumors in gynecology, with high incidence rate and mortality. It has become the main cause of death among tumors of the female reproductive system worldwide.^[6]^ The treatment of cervical cancer (CC) includes surgery, radiotherapy and chemotherapy. Patients with early CC can choose radical surgery or radical radiotherapy only. The therapeutic effects of the 2 are equivalent, and the 5-years survival rate and mortality are similar.^[7]^ Radiotherapy includes external radiation (ET) and brachytherapy (BT), both of them are important components of radiotherapy for CC. And there are 3 main technologies for brachytherapy: intracavitary (IC), interstitial (IS), and intracavitary/interstitial (IC/IS) combination.^[8,9]^Radiotherapy can be selected for all stages of CC. Concurrent chemoradiotherapy based on cisplatin is used for middle and advanced CC including locally advanced CC.^[10,11]^ The treatment choice should be comprehensively considered according to the patient’s age, pathological type and stage.^[7]^

Meningioma is the most common primary central nervous system tumors, and approximately half of them are benign tumors.^[12]^ Brain magnetic resonance imaging (MRI) is the radiological gold standard in the diagnosis of meningioma. The choice of the treatment mainly depends on the WHO grade of meningioma. However, surgery is still the main treatment. In recent years, more and more studies have confirmed that stereotactic radiosurgery (SRS) can be used in the treatment of meningioma^[13]^, achieving a good therapeutic effect without evident side effects. In general, the choice of the treatment should be comprehensively considered according to the patient’s medical history, clinical manifestation, histological grade and lesion location.

## 2. Case report

A 59-years-old female was admitted to the hospital due to vaginal bleeding for 1 month. She had a history of GBS, which caused the paralysis of the lower extremities. She suffered from hypertension for 5 years and diabetes for 3 years, but blood pressure and blood sugar at the moment of hospitalization were acceptable. After being brought to the ward using the wheelchair, the physical examination revealed stable vital signs, a grade II muscle strength of the right lower limb, a grade I muscle strength of the left lower limb, negative pathological signs and a soft lower abdomen, no tenderness and rebound pain, and no other positive signs.

**Figure 1.**
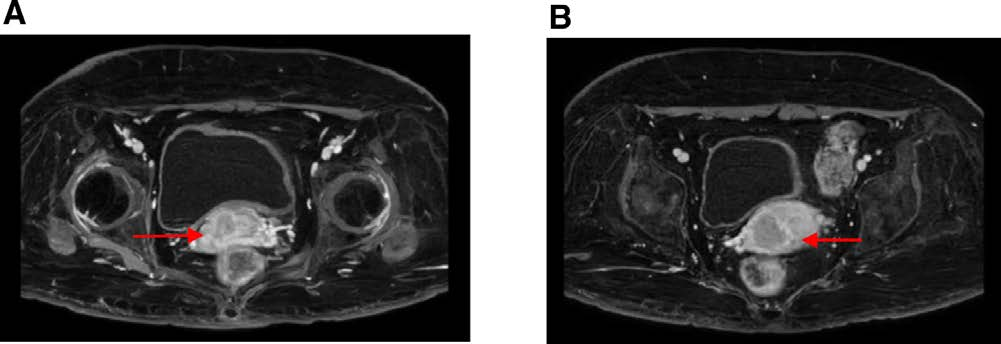
Pelvic contrast-enhanced MRI, showing the presence of uneven enhancement mass (arrow) in the right part of the cervix(A) and the posterior part of the uterine body(B), some parts of the cervical stroma are discontinuous, and the parauterine space on both sides is clear (before radiotherapy for CC). CC = cervical cancer, MRI = magnetic resonance imaging.

**Figure 2.**
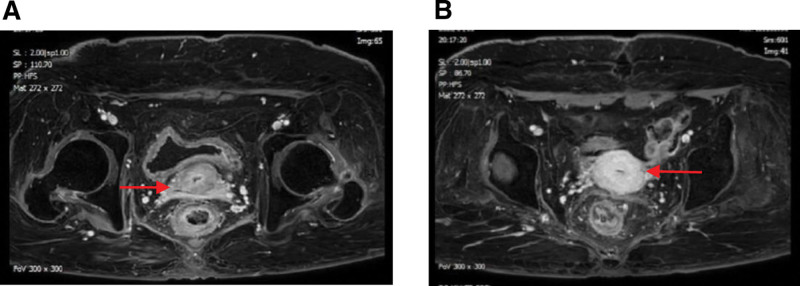
Pelvic contrast-enhanced MRI, showing the disappearance of the inhomogeneous enhancement mass (arrow) in the right part of the cervix(A) and the posterior part of the uterine body(B), the uterus basically return to the normal shape, and the bladder and rectal wall are thickened (after radiotherapy for CC). CC = cervical cancer, MRI = magnetic resonance imaging.

**Figure 3.**
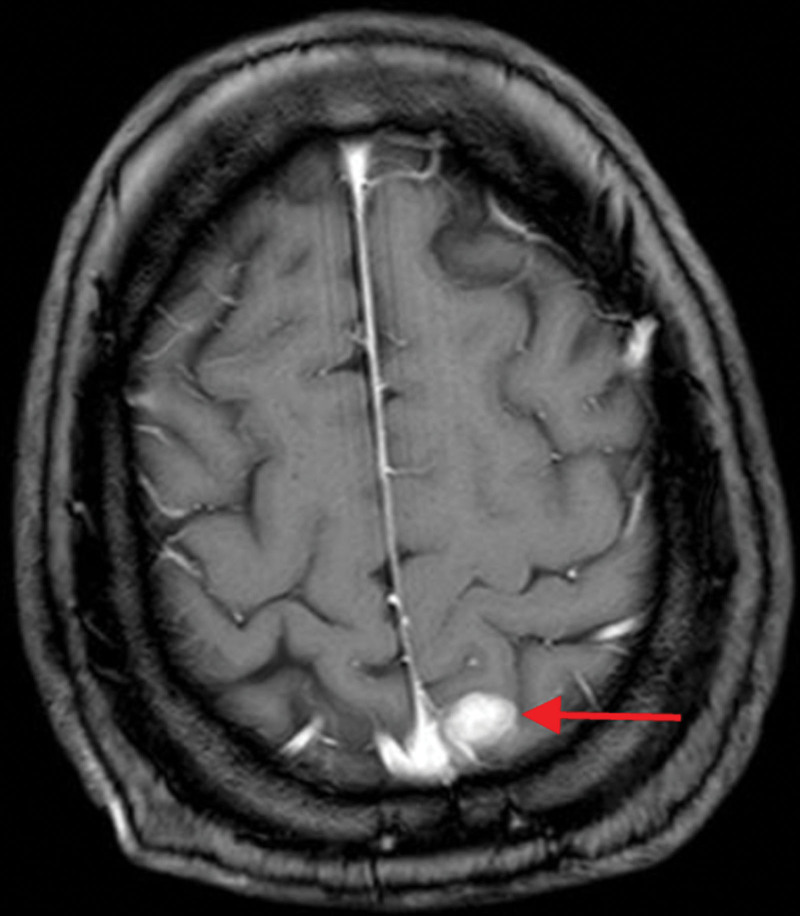
Enhanced MRI of the head showing the presence of an enhanced nodule (arrow) adjacent to the falx cerebri in the left parietal lobe (before SRS). MRI = magnetic resonance imaging, SRS = stereotactic radiosurgery.

**Figure 4.**
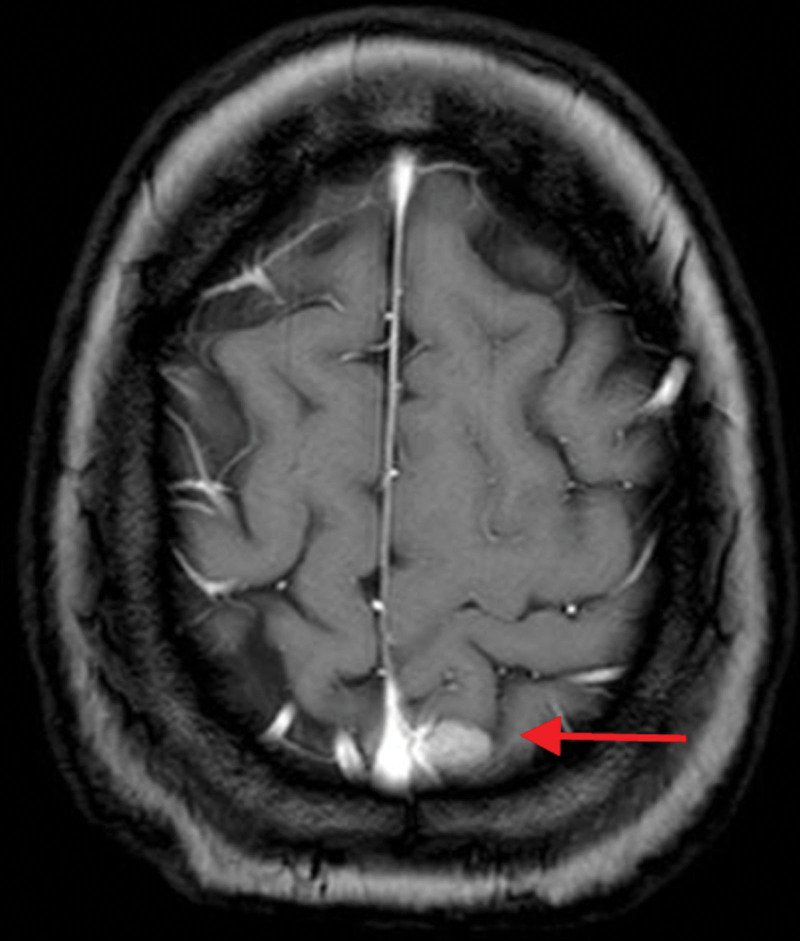
Head enhanced MRI reexamination showing a weakened enhancement of the nodule and no significant change in size (nearly 2 months after SRS). MRI = magnetic resonance imaging, SRS = stereotactic radiosurgery.

The gynecological examination revealed the presence of bloody secretions in the vagina and a mass of approximately 3 cm in diameter in the cervix. The surface of the mass was ulcerated, hard, and the mobility was poor. No palpable and evident abnormalities were observed in the uterus, bilateral adnexa and parauterine region. The cervical biopsy confirmed cervical squamous cell carcinoma. Pelvic MRI showed the presence of equal signal lump (plain scan) in the right part of the cervix and the posterior part of the uterine body. After enhancement, an uneven enhanced mass with a size of approximately 2.7*3.2*3.5 cm was found, some parts of the cervical stroma were discontinuous, and the parauterine space on both sides was still clear. The mass occupying the right part of the cervix and the posterior part of the uterine body was considered as CC. Brain enhanced MRI showed the presence of an enhanced nodule with a size of approximately 0.8*1.0*0.9cm in the left parietal lobe, and it was more likely to be considered as meningioma than metastatic tumor. No positive findings were obtained by the single photon emission computed tomography (SPECT) and chest and abdomen enhanced computed tomography (CT). The laboratory examination of the blood revealed the presence of the squamous cell carcinoma antigen: 2.82 ng/ml (normal reference range: 0–1.5 ng/ml), carbohydrate antigen 50: 26.05 U/ml (normal reference range: 0–25 U/ml), glycosylated hemoglobin: 7.0% (normal reference range: 4.5%–6.4%), while the levels of other tumor markers including CA125, CEA, AFP and CA153 were within the reference range. The clinical diagnosis was the following: Cervical squamous cell carcinoma (IB2 stage, FIGO 2018); Left parietal meningioma? 3. GBS; Hypertension; Type 2 diabetes mellitus. The patient’s condition was complex, and the consultation of the literature did not yield any relevant case reports and related guidelines for reference, which resulted in great difficulties to perform an appropriate treatment. The multi-disciplinary team (MDT) consultation led to the conclusion that the intracranial tumor was probably meningioma, and giving that GBS was stable, although tumor treatment or other factors can lead to GBS recurrence, tumor treatment, including radiotherapy and chemotherapy, could be still an option. After a discussion with the patient and her family, both the patient and family members refused the choice of surgical treatment. Thus, SRS was performed for the intracranial tumor, with a prescription dose of 12 Gy/1F (the prescription dose was placed on the isodose curve of 50%). Then, radical radiotherapy for cervical squamous cell carcinoma was started 4 days after the treatment, and cisplatin chemotherapy was administered once a week. The chemotherapeutic regimen was represented by an intravenous infusion of cisplatin 40 mg, once a week. Three-dimensional conformal radiotherapy (3D-CRT) was performed to the pelvic cavity, and the target area included the whole uterus, part of the vagina, periuterine and pelvic lymph node drainage area avoiding the bladder, rectum, femoral head and other dangerous organs. The prescribed dose was 50.4 Gy/28F. After the external irradiation, the patient rested for 1 week, and then, the intracavitary radiotherapy for CC started. The prescribed dose was 28Gy/7F, twice a week. During this period, weekly chemotherapy was completed 5 times (Table 1) (due to the severe gastrointestinal reaction after the first chemotherapy, the cisplatin dose of the last 4 chemotherapy was reduced to 30 mg), vaginal flushing was performed twice a week, and the overall side effects were acceptable. At 1 year follow-up the CC almost disappeared (Fig. 1, 2), and the meningioma were stable (Fig. 3, 4); the patient was generally in good condition, and both the patient and her family members were satisfied with the treatment results. In the last telephone follow-up recently, the patient was also in good condition without special discomfort. Evaluation of curative effect of CC after treatment: complete remission (CR), evaluation of curative effect of meningioma: stable disease (SD), according to Response Evaluation Criteria in Solid Tumors (RECIST). And GBS did not recur.

**Table 1:** Timeline about important milestones related to diagnoses and interventions.

Time	Important milestones	Result
2019-04-30	Cervical Biopsy	Cervical squamous cell carcinoma was confirmed
2019-05-06	Pelvic Enhanced MRI	An uneven enhanced mass with a size of approximately 2.7*3.2*3.5 cm was found in the right part of the cervix and the posterior part of the uterine body
2019-05-06	Brain Enhanced MRI	An enhanced nodule with a size of approximately 0.8*1.0*0.9cm was found in the left parietal lobe, and It was more likely to be considered as meningioma
2019-05-08	MDT	Discuss diagnosis and treatment plan
2019-05-10	SRS	SRS was performed for meningioma
2019-05-14 to 2019-07-19	Radical Radiotherapy	3D-CRT for external irradiation and 2-dimensional intracavitary brachytherapy technology for intracavitary radiotherapy
2019-05-14	Concurrent Chemotherapy	weekly chemotherapy was completed 5 times with cisplatin
2019-05-28	Concurrent Chemotherapy	
2019-06-04	Concurrent Chemotherapy	
2019-06-11	Concurrent Chemotherapy	
2019-06-18	Concurrent Chemotherapy	

3D-CRT = Three-dimensional conformal radiotherapy, MDT = multi-disciplinary team, MRI = magnetic resonance imaging, SRS = stereotactic radiosurgery.

## 3. Discussion

GBS is a rare but fatal autoimmune disease. Cervical cancer is one of the most common gynecological malignancies, and meningioma is the most common primary central nervous system tumor. However, cervical malignancie and meningioma are very rare in 1 patient with a history of GBS. No report is available after consulting the local and international literature. Since GBS in a stable stage can relapse during tumor treatment, our treatment decision-making was a challenge. No reliable guide and no relevant literature report was available as a reference. Finally, MDT consultation was performed and the treatment plan was decided. Thus, this work reports the diagnosis, treatment and treatment results of such patient.

The patient described in this article had a history of GBS. When CC combined with meningioma was found, the GBS condition was stable, leaving only the sequelae of decreased muscle strength in both lower limbs and no signs of recurrence. In terms of treatment, the treatment of CC and meningioma was relatively difficult and risky due to the previous GBS and multiple basic diseases because the tumor treatment, including the operation and the decline of resistance after operation, radiotherapy and chemotherapy, can be the cause of GBS recurrence.^[5]^ In severe cases, it may even involve respiratory muscles, making the treatment of the patient more complex and increasing the safety risk of the patient. No relevant case reports and references were found through literature review. Thus, it is difficult to make a decision for an appropriate treatment. Therefore, our hospital formulated the treatment strategy after MDT consultation.

Surgery is the main treatment for meningioma. The treatment choice depends on the patient’s medical history, severity of symptoms, histological grade, lesion location and risk/benefit ratio.^[14]^ SRS is an alternative to surgery,^[14–16]^ which gave good results in some specific patients, exerting very good therapeutic effects as the main treatment for small meningiomas^[13]^, which can be irradiated in a single dose or divided in different times. Stage IB2 (2018 FIGO) cervical squamous cell carcinoma which is less than 4 cm can be cured by radical surgery or radical RT.^[17]^ However, this patient refused to undergo surgery for CC and meningioma. Considering that SRS is precise radiotherapy, the treatment time is short, the brain tissue around the lesion can be well protected, and the side effects are mild, SRS was first performed for meningioma, so as to avoid some changes of the head lesion due to the long-time subsequent radiotherapy for CC, because the head lesion can not be completely excluded as a metastatic lesion. Of course, head SRS treatment can also be selected for isolated small head metastases,^[15]^ so the SRS treatment strategy for the head lesion is feasible. However, due to the lack of surgical treatment, the pathological tissue of the head lesion cannot be obtained, so this is the defect of this method. The patient’s head lesion was treated smoothly through SRS. Considering the patient’s limb movement disorder, the external irradiation of CC was performed using 3D-CRT technology, and the intracavitary radiotherapy was performed using 2-dimensional intracavitary brachytherapy technology, in order to reduce the time of the single radiotherapy, reduce the positioning error and organ displacement, and improve the patient’s comfort. The radiotherapy process was smooth, the side effects were acceptable and GBS did not relapse. After 1 year, the cervical and uterine tumor basically disappeared and the general condition of the patient was acceptable, the curative effect was satisfactory.

## 4. Conclusion

This report described a woman with a history of GBS who later developed CC and meningioma. The malignant tumor basically disappeared and the meningioma was stable after clinical treatment, and GBS did not relapse. As far as we know, this is the first report on such patients, providing some references in the treatment options of similar patients in the future.

## Author contributions

**Writing – original draft:** Kangli Deng.

**Writing – review & editing:** Yanxin Yu, Liben Ge, Kangyan Deng, Mo Zhang.
